# Leprosy: Comprehensive insights into pathology, immunology, and cutting-edge treatment strategies, integrating nanoparticles and ethnomedicinal plants

**DOI:** 10.3389/fphar.2024.1361641

**Published:** 2024-05-16

**Authors:** Neetika Kimta, Amin F. Majdalawieh, Gheyath K. Nasrallah, Sunil Puri, Eugenie Nepovimova, Klaudia Jomova, Kamil Kuča

**Affiliations:** ^1^ School of Biological and Environmental Sciences, Shoolini University of Biotechnology and Management Sciences, Solan, India; ^2^ Department of Biology, Chemsitry, and Environmental Sciences, College of Arts and Sciences, American University of Sharjah, Sharjah, United Arab Emirates; ^3^ Biomedical Research Center, Qatar University, Doha, Qatar; ^4^ Department of Chemistry, Faculty of Science, University of Hradec Kralove, Hradec Králové, Czechia; ^5^ Department of Chemistry, Faculty of Natural Sciences and Informatics, Constantine the Philosopher University in Nitra, Nitra, Slovakia

**Keywords:** leprosy, pathology, immunology, ethnomedicinal plants, secondary metabolites, nanotechnology

## Abstract

Mycobacterium leprae is the causative agent responsible for the chronic disease known as leprosy. This condition is characterized by dermal involvement, often leading to peripheral nerve damage, sensory-motor loss, and related abnormalities. Both innate and acquired immunological responses play a role in the disease, and even in individuals with lepromatous leprosy, there can be a transient increase in T cell immunity during lepromatous reactions. Diagnosing of early-stage leprosy poses significant challenges. In this context, nanoparticles have emerged as a promising avenue for addressing various crucial issues related to leprosy. These include combatting drug resistance, mitigating adverse effects of conventional medications, and enhancing targeted drug delivery. This review serves as a comprehensive compilation, encompassing aspects of pathology, immunology, and adverse effects of multidrug delivery systems in the context of leprosy treatment. Furthermore, the review underscores the significance of ethnomedicinal plants, bioactive secondary metabolites, and nanotherapeutics in the management of leprosy. It emphasizes the potential to bridge the gap between existing literature and ongoing research efforts, with a profound scope for validating traditional claims, developing herbal medicines, and formulating nanoscale drug delivery systems that are safe, effective, and widely accepted.

## Graphical Abstract

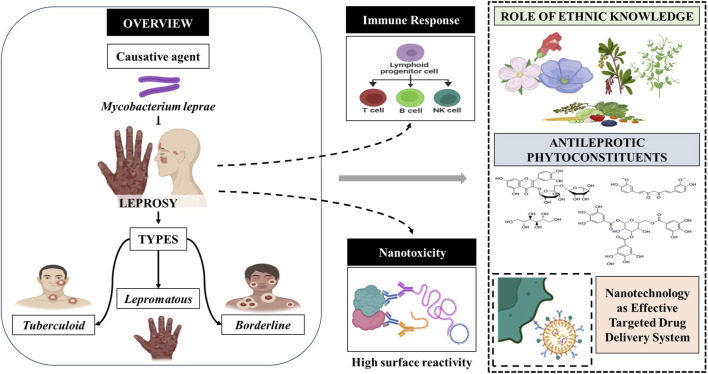

## 1 Introduction

Leprosy, while curable, remains a significant global health concern as it is not preventable. Two intertwined aspects characterize this complex disease. Firstly, a chronic mycobacterial infection triggers a wide spectrum of cellular immunological reactions in affected individuals. Secondly, it leads to peripheral neuropathy due to the infection and the subsequent immunological reactions. Leprosy is caused by *M. leprae*, an obligate intracellular bacterium that predominantly affects mucous membranes, nerves, and the skin (Talhari S. et al., 2015). In multibacillary individuals (MB), it can also impact various other parts of the body, including the bone marrow, eyes, internal organs, joints, lymph nodes, and the nose (Talhari C. et al., 2015). This condition, also known as Hansen’s disease, was named in honor of physician Gerhard Armauer Hansen (Hansen, 1875; Barbosa-Filho et al., 2007). Leprosy primarily spreads from person to person through contact with untreated individuals with a high bacillary index (Talhari S. et al., 2015). The clinical presentation of leprosy can vary, with the number of lesions depending on the severity of the disease (Talhari C. et al., 2015). A strong cellular immunity against *M. leprae* but a weak humoral immune response is typically associated with a few skin lesions. In contrast, individuals who cannot mount an efficient cellular-mediated response to *M. leprae* may present with numerous symmetrically distributed hypochromic lesions, known as Multibacillary individuals (MB), due to hematogenous dissemination of the bacilli. Patients with MB are considered the primary source of infection during the transmission cycle. While *M. leprae* has been found in breast milk, the main transmission mode is respiratory secretions and skin lesions (Kerr-Pontes et al., 2006; World Health Organization, 2012; Talhari C. et al., 2015). If not adequately treated, the disease can progress, leading to reactions that may result in severe damage to peripheral nerve trunks. This, in turn, causes sequelae of physical impairments, contributing significantly to the stigma associated with leprosy. Leprosy is characterized by nerve damage, disfiguring skin blisters, and gradual weakness. In the 1980s, *M. leprae* infection affected more than 5 million individuals globally, with the majority of cases found in Latin America, Asia, the Pacific Islands, and Africa (Barbosa-Filho et al., 2007). However, by the year 2020, the incidence of leprosy had decreased to less than 129,192 cases (Mushtaq, 2019). For almost 4000 years, people have been impacted by leprosy, which has left two to three million people permanently disabled (Ma et al., 2020). Brazil, in particular, has a high prevalence of leprosy cases, making it one of the countries with the second-highest number of cases worldwide. Although it has been historically seen as incurable, it is now treatable.

Leprosy is a valuable human model for examining host responses to intracellular infections, particularly those affecting peripheral nerves. Advances in medical science now allow for the analysis and diagnosis of leprosy patients using well-identified pathogen antigens. This includes glycolipids, proteins, peptides, and phenolics antigens, which can be detected in antibody and T-cell assays. Leprosy can be effectively treated with a therapeutic approach known as multidrug therapy (MDT) (McDougall, 1984; Manca et al., 2012). This treatment regimen not only aids in the patient’s recovery but also eliminates the causative pathogen. One significant challenge in leprosy research is *M. leprae* do not grow in artificial media (Sharma et al., 2014). Along with this the limitations of a suitable animal model that can replicate the entire spectrum of leprosy. An ideal animal model would be one that contracts the disease without immunosuppression, exhibits human-like bacteriological and histological characteristics, and develops the illness. Animals with body temperatures below 37°C appear to be more susceptible to *M. leprae* infection, limiting the number of susceptible animal hosts (Truman et al., 2014). To circumvent the problem that the *M. leprae* cannot be cultivated *in vitro,* nine-banded armadillos (*Dasypus novemcinctus*) are the most appropriate armadillo species for leprosy research (Balamayooran et al., 2015). Recently, wild chimpanzees in two different forest preserves in the West African countries of Guinea Bissau and Ivory Coast were found to have lepromatous leprosy. This discovery highlights the importance of considering a wide range of animal models in research to gain a comprehensive understanding of diseases and their potential treatments (Hockings et al., 2021). Although, research investigations often focus on patients who have already developed clinical symptoms, making it challenging to distinguish early causal effects from well-established markers of acquired immunity (Teles et al., 2010). Additionally, the extended disease incubation period, which can range from months to several years, further complicates research efforts.

Ethnopharmacology is a therapeutic modality that deals with the use of plants, plant extracts, or plant-derived pure compounds to treat disease, which was originally discovered via the study of traditional treatments and indigenous people’s folk knowledge. The screening of medicinal herbs holds promise as a potential source of biodynamic chemicals with therapeutic benefits. Ethnobotanical studies conducted in various regions worldwide have gained significance in developing conservation and healthcare initiatives (Barbosa-Filho et al., 2007). Throughout history, numerous natural remedies have been derived from medicinal plants. Common plants like willow bark (*Salix* spp.), foxglove (*Digitalis purpurea*), quinine bark (*Cinchona officinalis*), and Madagascar periwinkle have served as the basis for many modern medications, including aspirin, digitalis, paracetamol, quinine, and vinblastine. These plants contain active constituents such as flavonoids, glycosides, tannins, alkaloids, coumarins, and vitamins. Consequently, these metabolites have a broad spectrum of therapeutic applications and can protect the human body by impeding the growth of pathogens at various stages of development (Mittal et al., 2014). Using herbal remedies in Ayurveda holds immense potential for developing novel pharmaceuticals and making a substantial global impact (Gupta et al., 2014).

Nanotechnology represents a multidisciplinary domain encompassing science, technology, and engineering, focusing on nanoscale materials (Lu et al., 2022). Nanoparticles, a central nanotechnology component, typically measure between 1 and 100 nm in size. These minuscule particles exhibit remarkable reactivity, enhanced stability, and impressive adsorption capabilities, primarily due to their small size and high surface area-to-volume ratio (Hoshyar et al., 2016). In biomedicine, nanoparticles have garnered substantial interest from the scientific community, primarily due to these exceptional physicochemical attributes (Chakraborty et al., 2022). Both organic and inorganic nanoparticles find a wide array of applications in the medical field, ranging from gene therapy and early diagnosis to medical imaging. Their ability to traverse the blood-brain barrier and exhibit a degree of selectivity towards amyloid plaques positions nanoparticles as promising candidates in pharmaceutics (Joshi et al., 2021). Nanotechnology has been harnessed to tackle various critical challenges in healthcare, including combating drug resistance, mitigating adverse effects associated with conventional medications, and addressing the intricacies of targeted drug delivery in leprosy treatment. As a result, nanotechnology is emerging as a pivotal therapeutic field (Mitchell et al., 2021).

**FIGURE 1:**
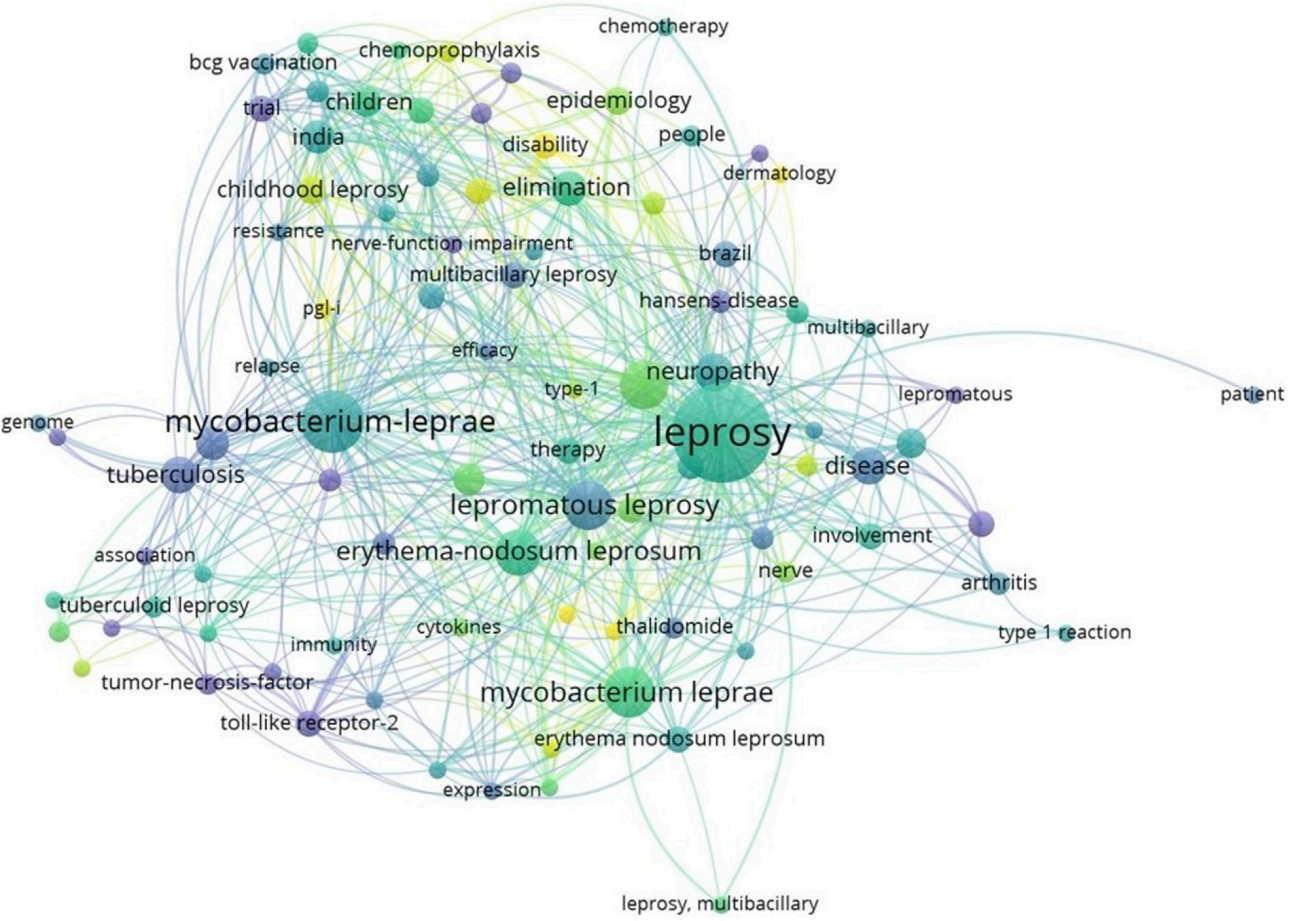
Representation of keyword co-occurrence map. A coloured circle represents each keyword, with the circle size indicating search intensity. (VOSviewer version 1.6.19).

Data from the Web of Science (WOS) spanning 2003 to 2023 were collected using the search term “leprosy.” This search yielded a total of 5668 peer-reviewed publications related to leprosy. The complete records and their associated cited references were exported in plain text format to conduct a comprehensive analysis. Subsequently, a keyword co-occurrence analysis was performed, and the results were visualized using VOSviewer version 1.6.19. As depicted in Figure 1, the term co-occurrence analysis unveiled strong connections between leprosy and various keywords, including *M. leprae*, epidemiology, tuberculosis, lepromatous leprosum, disease, and elimination.

The key objective of this review article is to represent a pioneering effort by encompassing a comprehensive spectrum of information, including pathology, immunology, therapeutic alternatives, ethnopharmacological insights, pharmacological data, and nanotechnology-based approaches. Despite the progress of the twenty-first century, leprosy continues to bear a social stigma, underscoring the crucial need for increased education, awareness, and research in this field. To compile this comprehensive review, we extensively searched the scientific literature across various databases using the keyword “leprosy”. These databases include, but are not limited to, Google Scholar, PubMed, Scopus, Plants for Future Database, Medicinal Herbs Database, Science Direct, Taylor and Francis, Elsevier, Springer, and Wiley Online Library. The review encompasses studies conducted between 1968 and 2023, spanning multiple domains such as ethnomedicine, pharmacology, and nanotechnology. In compiling this information, we ensured the accuracy of scientific plant names using the World Flora Online database.

## 2 Types of leprosy

**FIGURE 2:**
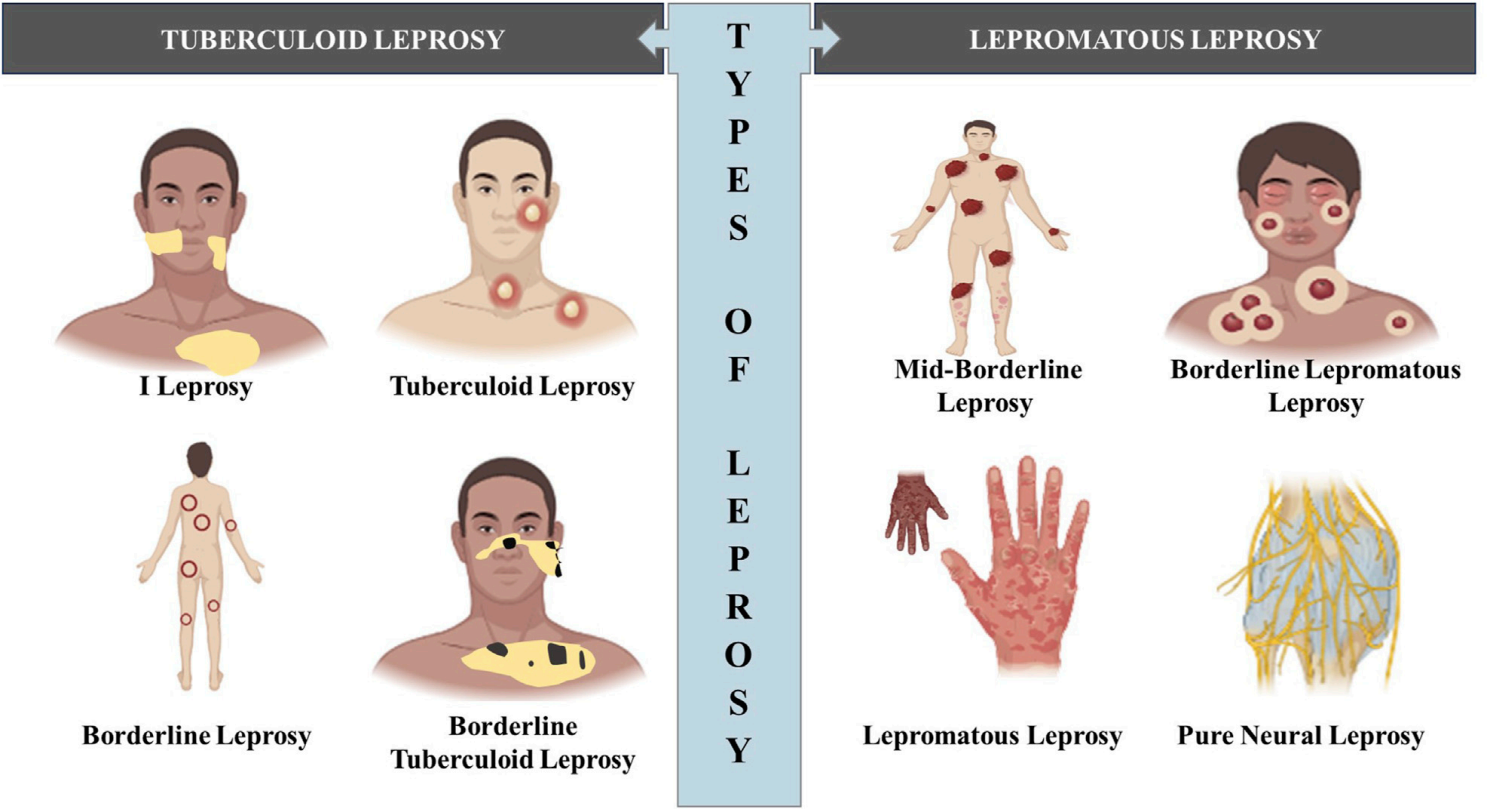
Diagram illustrating the various types of leprosy outlining the visual differences in appearance between the different types of leprosy. (Biorender).

In 1953, a classification system was proposed at the Madrid Congress, categorizing leprosy into four primary disease categories: lepromatous leprosy, tuberculoid leprosy, indeterminate leprosy, and borderline or dimorphous leprosy (Lockwood et al., 2007). This classification system was actually proposed at the 1953 International Leprosy Congress held in Madrid, hence the name and now is less frequently used. However, in 1962 and 1966, Ridley and Jopling, introduced a new categorization that took into consideration not only clinical characteristics but also histology, bacterial load, and the level of cell-mediated immune (CMI) response against *M. leprae*, as determined by the outcome of Mitsuda’s intradermal test (Ridley and Jopling, 1966; Singh et al., 2013a; Antunes et al., 2016). This updated classification system divides patients into five groups: tuberculoid leprosy (TT), characterized by elevated CMI representing the hyperegic pole, borderline-tuberculoid (BT), borderline-borderline (BB), borderline-lepromatous (BL), lepromatous leprosy (LL), which is poorly resistant (anergic) characterized by increased humoral immunity (Figure 2). While indeterminate leprosy (IL) was not part of Ridley and Jopling scheme, this clinical expression does not fit into the spectrum as there is no clear link between clinical and histological characteristics. The degree of CMI in this stage remains unclear, indicating an early stage of the disease (Britton and Lockwood, 2004).

### 2.1 Indeterminate leprosy

Most patients develop macular and hypopigmented lesions in the early stages of leprosy. This early clinical expression, IL is observed in individuals who have not yet developed CMI against *M. leprae* (Britton and Lockwood, 2004). These lesions are typically macules with smooth surfaces and do not exhibit scaling or itching. They are relatively small, usually up to 4 cm in size, and their color can vary from red to coppery, depending on the patient’s skin tone. Despite these lesions, patients still have bodily hair and normal sweating (Walker and Lockwood, 2007). One crucial characteristic of leprosy lesions is sensory impairment, often known as anesthesia. Patients with leprosy lesions frequently experience a lack of temperature perception, making it challenging to distinguish between hot and cold stimuli. Interestingly, skin lesions are often discovered after the onset of hyperalgesia, indicating that sensory abnormalities are part of the disease progression (Walker and Lockwood, 2007). It is worth noting that the number and severity of skin lesions can be influenced by the patients’ genetic predisposition and their CMI responses to *M. leprae*. Those with a strong CMI response typically exhibit fewer skin lesions, whereas the humoral immune response plays a weaker role (Walker and Lockwood, 2007).

### 2.2 Tuberculoid leprosy

Tuberculoid leprosy (TT) is characterized by one or a few small-sized lesions with distinct, raised borders, appearing as papules and plaques (Nunzi and Noto, 2008). These raised borders can indicate either peripheral expansion of the lesion or central healing (Gaschignard et al., 2016). Normal TT lesions typically exhibit reduced sweating, sparse body hair, and anesthesia. Initially, patients experience thermal anesthesia, followed by tactile and loss of pain sensation. In some cases of TT, patients may have areas of anesthesia without any changes in skin color or peripheral trunk nerve enlargement, which can occur with or without skin lesions (Gaschignard et al., 2016). Notably, lesions on the face may retain normal sensitivity due to the dense sensory innervation in this area, compensating for the damaged nerves (Britton and Lockwood, 2004). In the early stages of TT, patients may observe macules. In light-skinned individuals, these macules can appear erythematous or coppery, although they may appear uniformly hypopigmented. Due to a lack of sweating (anhidrosis), these macules have a dry surface and a rough texture. Over time, papules may develop at the edges of the macules, resulting from the robust CMI response associated with TT (Pfaltzgraff and Bryceson, 1985).

### 2.3 Borderline leprosy

According to the Ridley and Jopling classification, most leprosy patients fall within the borderline category (Singh et al., 2013a; Antunes et al., 2016). The category encompasses patients with varying degrees of disability, often due to significant peripheral nerve involvement, which can affect numerous nerves (Britton and Lockwood, 2004). One defining characteristic of the borderline group is instability. Without proper treatment, borderline patients may progress to lepromatous leprosy and eventually manifest the characteristic clinical features of this disease. Furthermore, patients can experience upgrades or downgrades in their classification during or after therapy. Borderline individuals often undergo reversal reactions, which may or may not be related to treatment. These reactions are typically marked by worsening skin lesions and nerve involvement and can lead to paralysis if left untreated. (Ramos-e-Silva and Rebello, 2001).

### 2.4 Borderline-tuberculoid leprosy

In borderline-tuberculoid leprosy (BT), the skin lesions share some similarities with those observed in tuberculoid leprosy but tend to be more numerous, ranging from 10 to 20 in number. These lesions are typically larger than those seen in TT leprosy. A notable feature of BT leprosy is the presence of satellite lesions, which can vary in color, ranging from hypochromic to reddish. These smaller lesions often appear adjacent to larger ones or extend like finger-like projections into the normal skin from the borders of plaques or macules. Within the same patient, BT leprosy lesions can exhibit size, shape, and color variations. Skin lesions in BT leprosy can become enlarged or ulcerated, particularly due to type 1 reactions, which are common in this form of disease (Pfaltzgraff and Bryceson, 1985; Britton and Lockwood, 2004). Nerves are heavily involved in BT leprosy reactions, making immediate treatment crucial to prevent permanent deformity and disability. In some cases, only anesthetic macules and nerve enlargement may be observed.

### 2.5 Mid-borderline leprosy

BB leprosy is characterized by infiltrating plaques that come in varying sizes. These plaques have a unique appearance, with a central area of spared skin, usually hypochromic, a well-defined inner edge, and a less distinct outer edge that invades parts of normal skin (Britton and Lockwood, 2004). This combination of lesions creates a pattern often described as resembling Swiss cheese. These characteristic lesions are typically found alongside other skin abnormalities such as macules, plaques, papules, and nodules. BB leprosy is also associated with disseminated reddish-coppery lesions that are symmetrically distributed on the body (Pfaltzgraff and Bryceson, 1985). BB leprosy represents the rarest and most unstable part of the leprosy spectrum. It tends to progress rapidly towards either the TT or LL polar forms, and patients with BB leprosy may experience a range of nerve involvement.

### 2.6 Borderline lepromatous leprosy

The early indicators of BL leprosy are hypopigmented macular lesions. These lesions initially appear symmetrically distributed in the affected individuals. Over time, these macules enlarge, become erythematous (red), and infiltrate deeper in the skin tissue. Their borders become irregular and encroach upon the surrounding healthy skin (Ramos-e-Silva and Rebello, 2001). As the disease progresses, these lesions gradually penetrate more extensive areas of the skin. Some patients with BL leprosy may develop lesions resembling plaques, papules, and nodules, which can mimic the clinical presentation of lepromatous leprosy (Gaschignard et al., 2016). In most BL cases, there is evidence of peripheral nerve involvement, although nerve sensitivity is less common than in BT leprosy. However, it is important to note that significant nerve damage can occur during reactions associated with BL leprosy (Gaschignard et al., 2016).

### 2.7 Lepromatous leprosy

Patients unable to mount an efficient CMI response to *M. leprae*, leading to the hematogenous dissemination of the bacteria, may present with numerous, symmetrically distributed hypochromic lesions. Without treatment, these patients progress to a non-resistant form of leprosy known as polar lepromatous leprosy (LL). It is worth noting that LL can also result from lack of treatment of BB and BL (Britton and Lockwood, 2004). Lepromatous leprosy is classified into two clinical subtypes: subpolar (LLs) and polar (LLp). Patients with LLs exhibit a lesser degree of anergy than those with LLp, and they tend to achieve bacteriological negativity more rapidly following treatment. Some patients initially presenting clinical expressions of LL may experience type 1 responses (Kumar et al., 2014). In LLs, the macules, nodules, and plaques have well-defined margins. Conversely, extremely anergic patients are more likely to develop LLp, characterized by diffused skin infiltration with fuzzy, indistinct margins (Gaschignard et al., 2016).

In certain cases, the hypochromic lesions initially appear on the skin, and over time, they can gradually infiltrate large body areas. Without treatment, the affected areas may experience spreading erythema, increased skin penetration, and a reduction in the appearance of wrinkles (Córsico et al., 1994). Hair loss is a common consequence in the areas that have been infiltrated. The loss of eyebrows typically begins at the outer ends, resulting in a distinctive condition known as madarosis. Some patients may also experience the loss of eyelashes (Pfaltzgraff and Bryceson, 1985). As skin infiltration deepens, skin folds become more prominent, leading to the characteristic clinical feature of facies leonina. Additionally, the hands and feet may show signs of infiltration, causing the skin to appear sensitive and shiny. On the infiltrated skin, papules and nodules may gradually develop, either individually or in groups. It is worth noting that certain body areas, such as the head, axillae, midline of the back, perineum, groin, and other warmer regions of the skin, are less affected than the rest of the body (Córsico et al., 1994). As the condition worsens, peripheral nerves may expand, leading to the loss of sensation in the hands, feet, and other affected areas. These developments can potentially result in disabilities (Córsico et al., 1994; Garbino et al., 2022).

### 2.8 Pure neural leprosy

Pure neural leprosy (PNL), often called neural leprosy, is an exceedingly rare variant of leprosy, primarily affecting one or more peripheral nerves (Garbino et al., 2022). According to consensus, individuals presenting one or two enlarged nerves are classified as having paucibacillary (PBL) leprosy. In contrast, those with more than two affected nerves are categorized as having MB leprosy. The indications of nerve damage in PNL encompass sensory loss, muscle weakness, diminished sweating ability, and swollen or painful nerves. Another indications of PNL include motor impairment by developing weakness, muscle wasting (atrophy), and paralysis in the affected limbs due to damage to the motor nerves. Furthermore, enlargement of peripheral nerves, known as nerve hypertrophy or neuritis is also n indication of PNL. This enlargement is caused by inflammation and infiltration of the nerves by immune cells in response to the infection (Khadilkar et al., 2021). Identifying whether a nerve is swollen is not always straightforward. For patients with challenging-to-diagnose PNL cases, procedures like biopsy, involving fine needle aspiration and PCR or electroneuromyography, can be particularly valuable whenever feasible (Flageul, 2012). Recent studies indicate that asymptomatic leprosy cases with nerve swelling, with or without pain, can be identified and treated early through the use of high-resolution ultrasound. Specifically, individuals who fall outside the normal range for nerve thickness in the principal nerves affected by leprosy can be diagnosed and treated early. By identifying abnormal nerve thickness in these individuals, even in the absence of symptoms, healthcare professionals can diagnose silent peripheral neuropathy associated with leprosy and initiate early treatment (Voltan et al., 2023).

## 3 Pathology of leprosy

Research on the skin lesions caused by leprosy has contributed to a better understanding of its immunopathology (Fabel et al., 2019; Ma et al., 2020). In TT granulomas display scattered CD4^+^ T cells within the epithelioid cells and CD8^+^ T cells around the periphery. In the case of LL, B lymphocytes are notably scarce. Leprosy granulomas have been observed to contain an abundance of γδT lymphocytes (Ma et al., 2020). It has been shown that TT and IL response skin lesions exhibit higher levels of CD1 molecules (Lewinsohn and Lewinsohn, 2019). Langerhans cells seem to migrate into the dermis in T-lep granulomas, whereas Langerhans cells are diminished in LL (Fabel et al., 2019). This migration may facilitate the transfer of antigens from the skin to the appropriate T cells for presentation. Dermal lesions show increased CD4^+^ and IL-17-producing cells during reactional stages. CD4^+^ T cells were also present during erythema nodosum leprosum (ENL) in earlier lymphophenic LL lesions (Fabel et al., 2019). Local release of IgG and IgM has also been demonstrated (Qiong-Hua et al., 2013). Th1 and Th2 cytokines are expressed in T-lep and L-lep granulomas, respectively. Both reversal and ENL reactions exhibit a polarization toward the Th1 type (Moraes et al., 2001; Pandhi and Chhabra, 2013). Studies involving the injection of recombinant IFNγ into dermal lesions have shown faster clearance of bacilli compared to a control group that received only MDT, further supporting the role of IFNγ in eliminating leprosy bacilli (Nathan, 2020). It was noted that the injection sites of the lesional cells exhibited elevated levels of NO radicals, which may be responsible for bacterial death and tissue damage (Schmitz et al., 2019). The effectiveness of cytokines, compared to a combination of various anti-leprotic drugs, was evident as these changes were observed as early as 3 weeks following IFNγ injection. A multicenter study with long-term follow-up study identified TNFα, iNOS, and TGFβ in the skin of patients with type 1 reactions (Lockwood et al., 2011).

### 3.1 Reactions of leprosy

**FIGURE 3:**
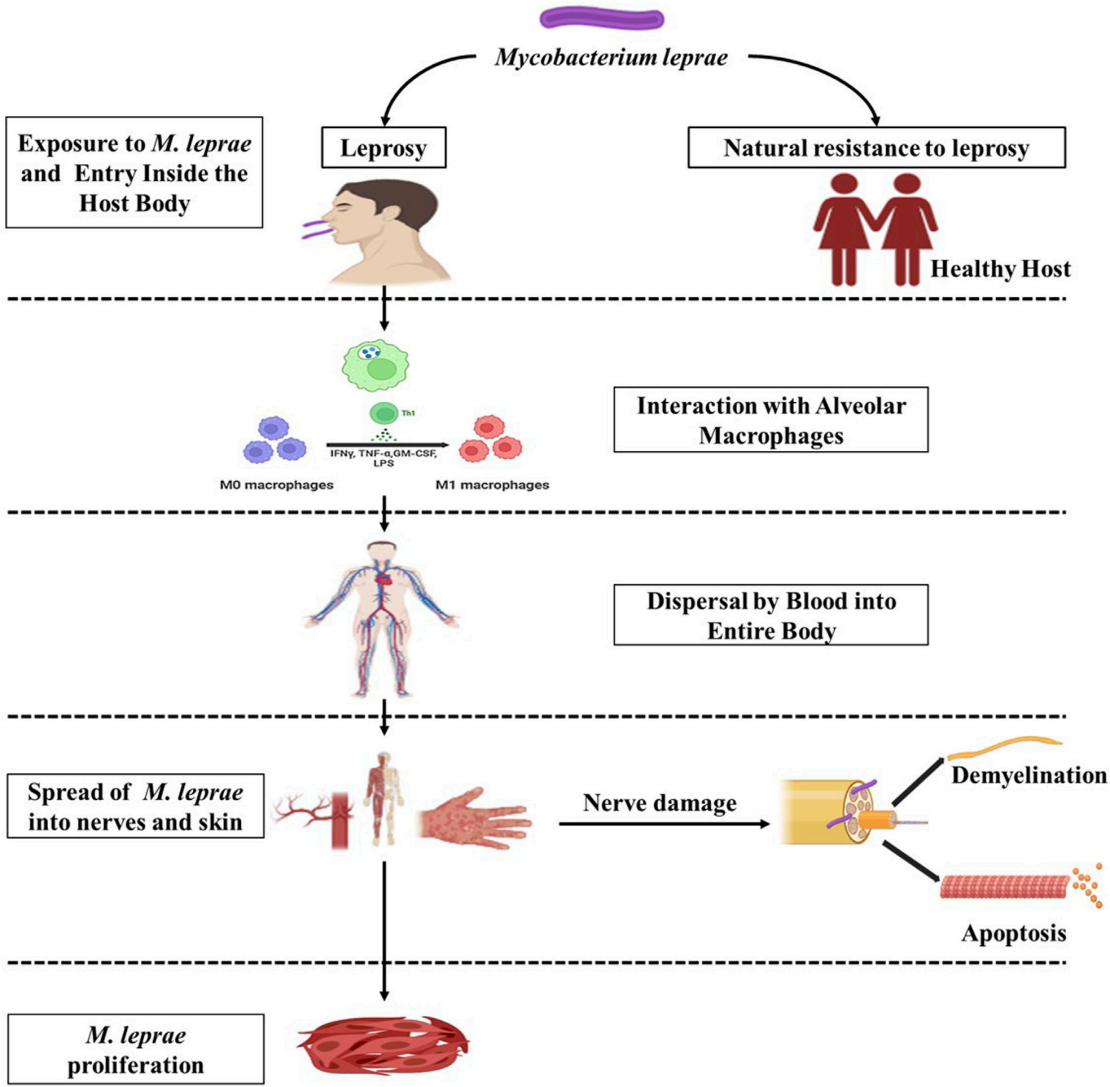
Schematic representation depicting the interaction between the host and *M. leprae*. (Biorender).

Leprosy reactions, characterized by acute inflammation, vary in frequency and severity depending on the country, affecting 10%–20% of Indian patients and 40%–50% of patients from Latin America and Mexico, with the latter group experiencing more severe forms of the reaction. These reactions often affect nearby peripheral nerves, leading to the need for prompt medical attention to alleviate excruciating pain and prevent nerve damage and deformities. There are two primary clinical categories: Type 1 or reversal reactions (RR), primarily seen in individuals with borderline leprosy (e.g., BT, BB, and BL), which are confined to the dermal patch and nearby nerves, and Type 2 or erythema nodosum leprosum (ENL), which predominantly occurs in lepromatous leprosy (L-lep). In Type 1 reactions, an inflammatory response occurs in the skin and nerves affected by the disease due to T cell-mediated responses triggered by *M. leprae* (Figure 3). This response involves an increased release of proinflammatory cytokines and enhanced lymphoproliferation in response to *M. leprae* antigens (Moraes et al., 2001; Pandhi and Chhabra, 2013). Recent data also suggest elevated serum levels of IP-10, an IFN-γ-induced chemokine, during reversal reactions (Stefani et al., 2009; Scollard et al., 2011). A TLR2 mutation has been linked to reactions of type 1 leprosy. (Bochud et al., 2008). Initially, it was believed that immune complex deposition in arteries, similar to the Arthus reaction, was responsible for Type 2 or ENL reactions (Garbino et al., 2022). However, this deposition is not consistently evident, and conventional immune complex disease is not a typical feature of ENL. ENL lesions have been found to contain CD3^+^ CD4^+^ T cells and transient antigen-specific T cell activation, as well as the production and release of IL-12 and IFN-γ. In the management of reactions in leprosy, particularly reversal type 1 reactions (RR) and type 2 erythema nodosum leprosum (ENL), corticosteroids and thalidomide are indeed commonly used to prevent inflammation from causing further nerve damage and to alleviate symptoms. They help suppress inflammation and reduce tissue damage associated with these reactions (Lockwood, 1996). Notably, skin tests remain negative in LL patients, indicating a failure to develop delayed-type hypersensitivity (DTH) in the skin. Some studies have shown an increase in IL-4, IL-6, and IL-8 (chemotactic for neutrophils), consistent with histological evidence of neutrophil infiltration in ENL lesions (Moraes et al., 2001). Research involving the recombinant protein LSR250 and its peptides in ENL revealed that hidden *bacillus* locations may become exposed and recognized by L-lep. Patients recognized specific sequences both during and before ENL (Talhari C. et al., 2015). Injecting IFN-γ intra-lesionally or inducing a delayed-type response with purified protein derivative (PPD) also induced ENL (Nathan, 2020). Thalidomide has been shown to suppress neutrophil apoptosis and pro-inflammatory TNFα in steroid-resistant ENL patients. Additionally, cyclosporine provides clinical benefits in ENL by inhibiting IL-2 and other cytokines. In conclusion, T-cell responses emerge abruptly in L-lep patients experiencing reactions, and these responses persist long after clinical symptoms begin to fade (Saini et al., 2013).

### 3.2 Nerve damage

Leprosy is characterized by the involvement of peripheral nerves, resulting in sensory loss, and it appears to involve both immunological and non-immunological processes. The primary target of *M. leprae* is the Schwann cell (SC), which acts as its host and main target. In laboratory culture systems using Schwannomas, it was demonstrated that the presence of phenolic glycolipid 1 (PGL-I) is crucial for the internalization of the microbe and subsequent demyelination (Rambukkana, 2004). Schwann cells keep the bacilli alive for an extended period while also defending against their destruction. The nerve damage in leprosy is primarily caused by immunological reactions to Schwann cells infected with *M. leprae*. Human Schwann cells in long-term cultures have been shown to express MHC class I and II, ICAM-1, and CD80 surface molecules, all involved in antigen presentation. Recent research has shown that both native and recombinant *M. leprae* proteins and peptides are processed and presented to T cells by human Schwann cells (Spierings et al., 2000). Activated T cells subsequently kill the infected Schwann cells. Leprosy nerve lesions contain stable and reactional forms of TNFα and TNFα mRNA, which may contribute to demyelination. Furthermore, nerve injury in leprosy may be caused by TLR2 on Schwann cells (Oliveira et al., 2003). Nerve injury in leprosy is contributed to by DTH reactions observed in type 1 reactions, as well as the deposition of local immune complexes and activation of complement in chronic ENL. In culture systems, contact-dependent demyelination has been observed without immune cells, suggesting that non-immune mechanisms may play a role in the early stages of nerve infection (Rambukkana, 2004). While non-myelinating Schwann cells are densely colonized by *M. leprae*, myelin-associated Schwann cells appear comparatively free of infection. Earlier ultrastructure studies on nerves from leprosy patients demonstrated the presence of the bacilli inside the Schwann cells of myelinated axon (Cabral et al., 2022).

## 4 Immunology of leprosy

In 1954, Mitsuda conducted a groundbreaking experiment demonstrating that intradermal injection of deceased *M. leprae* bacteria elicited a skin reaction after 3–4 weeks, characterized by erythema and edema at the injection site. Importantly, only patients with TT displayed these reactions, while patients with LL did not. This discovery implied that an individual’s immune response to the bacterium was necessary to develop an inflammatory reaction. Subsequently, Dharmendra conducted research showing that a lipid-free soluble component derived from leprosy bacilli could also induce such a reaction, but in a shorter time frame, typically occurring between 48 and 72 h. The temporal kinetics of the Dharmendra test align with the characteristics of a delayed-type hypersensitivity reaction, while the Mitsuda test assesses the granulomatous response. Both tests are still used today to assess immunological status, even though neither is specific to leprosy. In contrast to individuals with L-lep who may have antibodies but limited T cell function, histopathological features, skin tests, and immunological studies indicate that T-lep patients possess T cell immunity and exhibit DTH responses to the pathogen (Nathan, 2020).

### 4.1 Innate immunity

**FIGURE 4:**
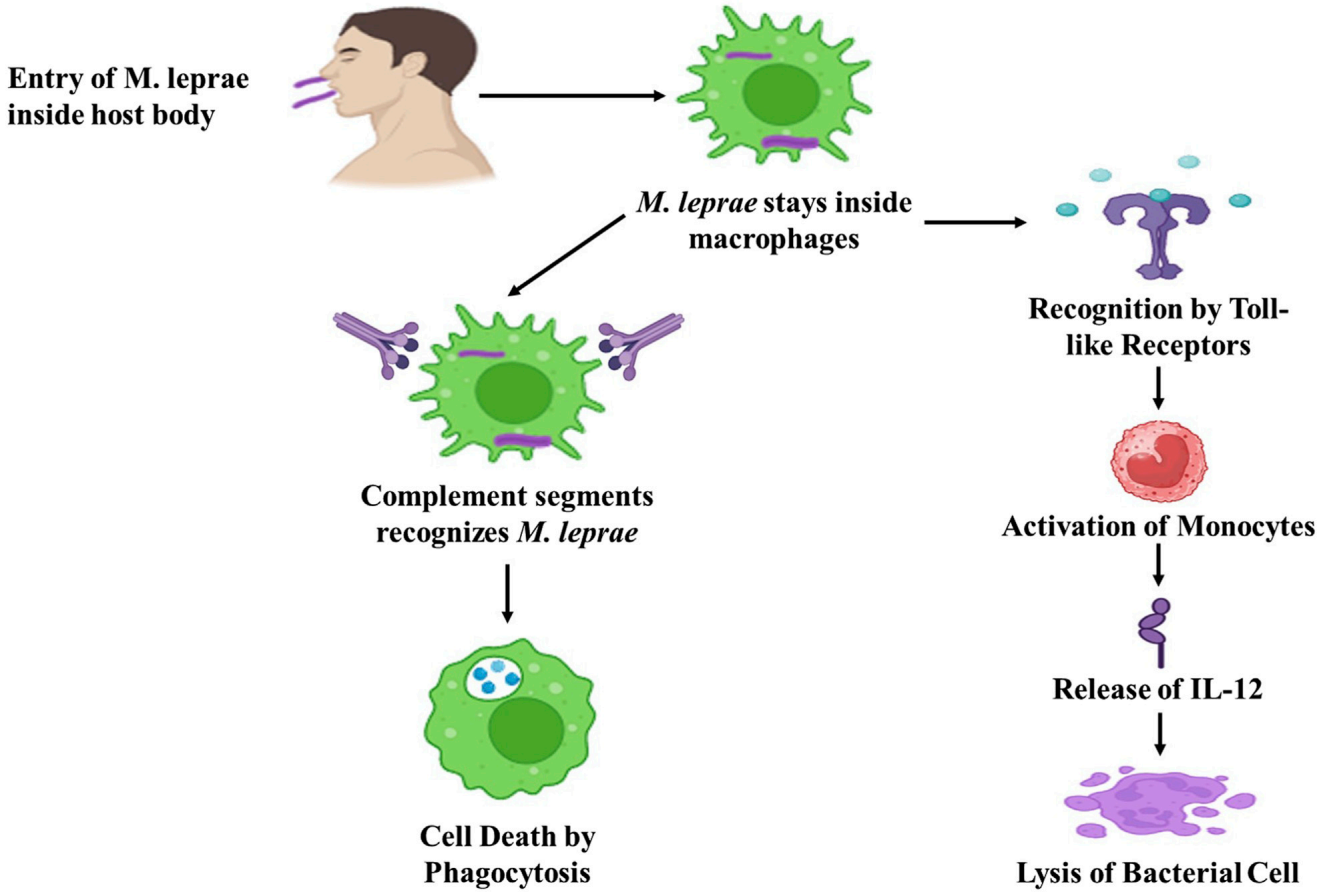
Diagrammatic representation of innate immune response against *M. leprae.* (Biorender).

The innate immune response is crucial to the body’s defense against infections and external substances. In the context of leprosy, dendritic cells, Schwann cells, and macrophages serve as entry points and host cells where *M. leprae* enters and resides. The first step in the pathogen’s intracellular lifestyle is the entry process, and *M. leprae* employs various mechanisms to infiltrate host cells. In leprosy, phagocytosis is facilitated by receptors that recognize complement fragments, such as CR1, CR3, and CR4. PGL-I, a cell wall lipid specific to *M. leprae*, is recognized by Complement 3.6. Complement and toll-like receptors (TLRs) are critical for detecting microbial pathogens found in macrophages and dendritic cells. Many pathogens are recognized by these cells through generic molecular pattern recognition (Figure 4). Within the family of TLRs, which bind to lipoproteins, TLR2 and TLR4 are responsible for recognizing the leprosy *bacillus*. When these receptors are activated, monocytes release IL-12, a cytokine that promotes the killing of the *bacillus* by proinflammatory cytokines. Cytokines like IFNγ and GM-CSF can increase the expression of TLR1, leading to the release of TNFα, which, in turn, induces inflammation. Notably, PGL-I, a glycolipid unique to *M. leprae*, induces the production of low levels of TNFα, IL-1, and IL-10, as well as negative regulatory molecules like MCP-1 and IL-1Ra (Teles et al., 2010; Manca et al., 2012).

### 4.2 Acquired immune response

**FIGURE 5:**
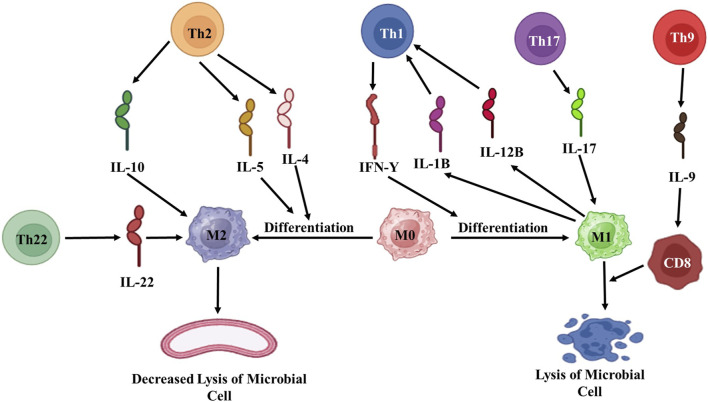
Diagrammatic representation of acquired immune response against *M. leprae.* Th: T helper; IL: Interleukin; M: Macrophages; CD: Cytotoxic T-cells. (Biorender).

The acquired immune response is a highly specialized interaction involving lymphocytes, dendritic cells, macrophages, as well as soluble components like antibodies produced by plasma cells. Such antibodies are responsible for capturing free microorganisms. In contrast, cytokines released by T cells can penetrate cell membranes to target intracellular pathogens (Figure 5). Interestingly, it has been observed that certain cytokines, such as IL-4 (a Th2 cytokine) and IL-10, play a negative regulatory role as they suppress the expression of TLR2 and cytokine production, which results from the interplay between the innate and acquired immune responses. In the context of leprosy, TLR1 and TLR2 are expressed more significantly in tuberculoid leprosy skin lesions, indicating their role in the immune response (Gautam et al., 2021).

### 4.3 T cell-mediated immune response

Skin tests made early detection of delayed type responses driven by divergent T cells in the leprosy spectrum possible. Early warning signs included T cell counts, PBMC nonproliferation in mitogen response, and unresponsiveness to *M. leprae* antigens in L-lep patients. While the reduced responses to T cell mitogens could be improved with medication, this specific unresponsiveness persisted for an extended period (Silva et al., 2022). Notably, the T cell response to other antigens, such as *M. tuberculosis*, remained unaffected in L-lep patients. This suggests that L-lep patients exhibit distinct antigen-specific unresponsiveness. Despite extensive research, no consensus exists on the underlying cause of this immunologic unresponsiveness in L-lep. This lack of reactivity, however, is believed to be due to peripheral tolerance rather than central tolerance or the loss of T cells specific to *M. leprae*. Antibiotic-mediated suppression was considered a potential factor, but this notion has lost support over time.

## 5 Conventional treatments of leprosy

### 5.1 First-line drugs

**FIGURE 6:**
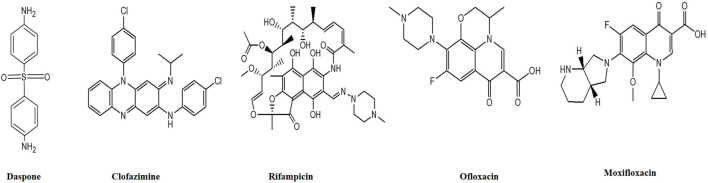
Chemical structures of anti-leprotic drugs. (ChemDraw).

Multi-drug therapy (MDT) is the cornerstone of modern leprosy treatment and consists of three drugs: Dapsone, Rifampicin, and Clofazimine (Figure 6). Detailed description of each of these medications is presented below.

#### 5.1.1 Dapsone (4,4-diaminodiphenylsulfone)

Before the introduction of Dapsone by Guy Faget as a therapeutic option for leprosy in 1946, the primary treatment for leprosy involved the intradermal injection of chaulmoogra or hydnocarpus oil, which is derived from the seeds of an herbal tree. These oils and their esters had weak anti-leprotic properties and were the mainstay of leprosy treatment (Rathod and Kalra, 2022). Dapsone is a bacteriostatic medication that functions by competitively inhibiting the enzymes dihydrofolate synthetase and dihydrofolate reductase, both of which are essential components of the folate production mechanism in *M. leprae* (Molinelli et al., 2019). Patients treated with Dapsone monotherapy typically experienced the complete eradication of bacilli within 3–6 months, although complete clinical regression often took 2–3 years. The healing process typically began with mucosal lesions, followed by the resolution of skin ulcers, clearance of nasal passages, reduced epistaxis (nosebleeds), and a decrease in foul-smelling nasal discharge. Regression of nodules and skin thickening occurred at a later stage. It is important to note that the response to treatment was very slow and often incomplete in cases involving nerve thickness, sensorimotor loss, and trophic ulcers. Patients with leprosy receiving Dapsone treatment needed special attention and protection to prevent burns and injuries, particularly to the eyes and extremities (Molinelli et al., 2019). Despite being generally well-tolerated, Dapsone is associated with certain well-known side effects. Dapsone the first efficacious medicine produced for the treatment of leprosy was first employed as a monotherapy treatment until the emergence of resistance in approximately 1960, which was subsequently attributed to mutations in the folP1 gene. Subsequently, the use of multidrug therapy (MDT) was suggested as a preventive measure against the development of drug resistance, replacing the use of monotherapy. It is important to note that dapsone hypersensitivity syndrome occurs in 0.5%–3.6% of people who are treated with this medication for different illnesses, including leprosy. This syndrome can be fatal in up to 10% of patients who have the specific HLA allele HLA-B*13:01 (Zhang et al., 2013).

#### 5.1.2 Rifampicin

Since its introduction in 1970, Rifampicin has been the only MDT component that exhibits bactericidal activity against *M. leprae*. Rifampicin achieves this by selectively inhibiting bacterial DNA-dependent RNA polymerase, thus halting the production of RNA (Campbell et al., 2001). Notably, Rifampicin is effective against bacteria resistant to Dapsone, making it a crucial component of leprosy treatment. In order to properly kill intracellular organisms, Rifampicin also functions by piercing bacterial cell membranes. Within 4–6 weeks of starting treatment, the morphologic index (MI) reaches zero. However, it takes longer for the bacteriologic index (BI) to decrease. Fortunately, the side effects are rare when Rifampicin is administered once a month. It is important to note that a mutation in the RpoB gene has recently emerged, leading to the development of resistance to Rifampicin. This resistance has become a significant concern, diminishing the usefulness and effectiveness of the medication in some cases (Chakraborty et al., 2022).

#### 5.1.3 Clofazimine

Clofazimine, a brick-red, fat-soluble crystalline dye, possesses bacteriostatic and anti-inflammatory properties. Its mode of action includes several biological targets. Clofazimine may prevent DNA from serving as a template for cellular processes. It can boost the production of lysosomal enzymes involved in cellular digestion (Kar and Gupta, 2015). Clofazimine is known to enhance the phagocytic activity of macrophages and other immune cells. It selectively attaches to mycobacterial DNA with a high content of GC (guanine-cytosine). Clofazimine is useful in treating type 2 leprosy responses (T2R) by increasing the production of prostaglandin E2 (PGE2), inhibiting neutrophil migration, and selectively suppressing Th1 responses (Moon and Joo, 2015).

Nuclear factor of activated T cells (NFAT) and nuclear factor κB (NFκB) promote the synthesis of interleukin 2 (IL-2), a key cytokine in type 1 leprosy reactions (T1R), which causes neuritis. It affects immune signaling pathways by blocking the potassium channel Kv1.3 and impacting the calcium-release triggered calcium channel oscillation frequency. This inhibition also extends to the calcineurin-NFAT signaling pathway (Ren et al., 2008). Notably, resistance to Clofazimine is extremely rare, likely due to its multiple mechanisms of action (George, 2020). However, the medication has a distinctive side effect as it accumulates in various tissues such as the skin, subcutaneous fat, liver, lungs, adrenals, kidneys, lymph nodes, and gastrointestinal tract. This accumulation leads to a characteristic crimson coloring of the skin and different discharges. It takes an average of 6–12 months for the body to eliminate the medication. After stopping Clofazimine, patients may require more than 3 years to return to their usual skin color (Kar and Gupta, 2015). It is important to emphasize that Clofazimine is not a substitute for the less expensive and more potent medication Dapsone, nor should it be used as a standalone treatment. Instead, it is typically part of a combination therapy regimen for leprosy treatment.

### 5.2 Novel drug treatment

The advancement of DNA sequencing techniques has led to the identification of numerous cases of resistance to first-line medications such as Dapsone and Rifampicin (Kar and Gupta, 2015). This highlights the importance of developing new medications and treatment strategies, especially for resistance or medication toxicity cases. While resistance is a relatively minor issue, it can potentially become a more significant problem in the future (World Health Organization, 2009). As a result, ongoing research is focused on developing newer medications, and treatment regimens are continuously reviewed and updated to address emerging challenges in patient management.

#### 5.2.1 Fluoroquinolone-based regimens (ofloxacin and moxifloxacin)

More than 2 decades ago, fluoroquinolone (FQ) antibiotics were first introduced as a treatment for leprosy. Early studies explored the use of a single-dose regimen consisting of Rifampicin, Ofloxacin, and Moxifloxacin (ROM) as well as a daily regimen of Rifampicin, Ofloxacin, and Moxifloxacin (RO) for 28 days. However, the current focus is on administering ROM (intermittent treatment) monthly for 12 months in the case of MB and 6 for months in the case of PB, especially when previous regimens have failed (Kar and Gupta, 2015). These newer regimens include both daily self-administration of medications and fully supervised treatment options.

#### 5.2.2 Uniform MDT

A uniform multi-drug therapy (U-MDT) approach involves providing the same treatment to all leprosy patients. PB and MB leprosy patients receive Rifampicin, Clofazimine, and Dapsone for 6 months. The Global Leprosy Programme (GLP) and the World Health Organization (WHO) have initiated a clinical trial to compare U-MDT with the traditional WHO-MDT regimens for treating both MB and PB leprosy (Kroger et al., 2008). In the U-MDT group, which consisted of 2094 PB patients and 1302 MB patients, there were 6 cases of recurrence, with 4 of them occurring among MB patients. According to Rao et al., 2009, the 6-month U-MDT regimen was well-tolerated and showed minimal therapeutic efficacy in PB leprosy. However, it was deemed too short a regimen to cure MB leprosy effectively.

#### 5.2.3 3 Accompanied MDT

The study conducted by Rao et al., 2009 found that the 6-month U-MDT regimen was well-tolerated but had minimal therapeutic effectiveness in treating paucibacillary (PB) leprosy. However, it was deemed insufficient in effectively curing multibacillary (MB) leprosy. To address these limitations, an approach known as Accompanied Multi-Drug Therapy (A-MDT) was introduced. A-MDT involves providing certain patients with a complete course of therapy during their initial visit to a leprosy clinic immediately following diagnosis. This approach is particularly recommended for populations living in challenging and hard-to-reach areas, such as border regions, urban slums, conflict zones, and migrant workers, as advised by the WHO (Kar and Gupta, 2015).

### 5.3 Challenges in treatment

To evaluate both humoral and cellular immunity, researchers actively identify and characterize antigenic biomarkers that trigger the host’s immune responses. While early studies relied on traditional techniques that involved extracting and purifying proteins from the bacteria, contemporary researchers now utilize the *M. leprae* genome sequences and bioinformatics tools to identify potential antigens. However, this approach has delayed early diagnosis (Sales et al., 2007). First-line anti-leprotic drugs have various side effects. For example, Dapsone can cause gastric intolerance and hemolytic anemia, especially in individuals with G6PD deficiency (Raman et al., 2009). Clofazimine may result in reddish-brown skin pigmentation (observed in 75%–100% of patients), ichthyosis, dryness (seen in 8%–28% of patients), erythroderma, acneiform eruptions, and monilial cheilosis (seen in <1% of cases) (Raman et al., 2009). Rifampicin has been associated with hepatotoxicity, thrombocytopenia, osteomalacia, and acute renal failure due to interstitial nephritis. Rifampicin can also lead to respiratory syndrome (characterized by dyspnea, cough, and fever) and flu-like syndrome (with symptoms like chills, shivering, fever, headache, and bone and joint pains), as well as various gastrointestinal problems such as nausea, vomiting, diarrhea, and abdominal pain (Raman et al., 2009). Clearly, while being effective against leprosy, these drugs often generate several side effects in the human body. Additionally, the emergence of drug-resistant bacteria is a growing concern.

The emergence of drug-resistant strains has become a significant global issue (Shah et al., 2007). In response to this scenario, the Global Leprosy Program of the World Health Organization (WHO) initiated a sentinel monitoring program to monitor medication resistance in leprosy. This initiative was initiated with an informal consultation on rifampicin resistance in leprosy, which took place in Agra, India, in 2006 (World Health Organization, 2007). Initially, rifampicin was given either alone or in conjunction with dapsone. In 1976, the first case of resistance to rifampicin was recorded. This occurred in a patient who had relapsed while being treated with rifampicin alone, after having previously been treated with dapsone (Jacobson and Hastings, 1976). Susceptible strains become resistant to rifampicin due to the occurrence of missense mutations at codons 438, 441, 451, 456, and 458. The amino acid alterations in the β subunit prevent the binding of rifampicin to RNA polymerase (Matsuoka, 2010). The occurrence of a missense mutation at codon 456, where the amino acid serine (TCC) is replaced by leucine (TCG), is more commonly detected in *M. leprae* isolates that are resistant to rifampicin (Williams and Gillis, 2012). A study conducted by Cambau and coworkers indicated that drug resistance in leprosy is a serious and increasing problem (Cambau et al., 2018). It highlighted the frequency of drug resistance for key medications used in leprosy treatment, including dapsone, rifampicin, and quinolones. Furthermore a study conducted by Neto and coworkers regarding alternative drug regimens for cases of MDT therapy failure not due to specific drug resistance markers (Neto et al., 2020). The study suggests the use of alternative drug regimens for cases of leprosy that do not respond to the recommended treatment regimen. These regimens include daily administration of moxifloxacin, clarithromycin, minocycline, and clofazimine. The study evaluates the effectiveness of this alternative regimen in cases where the standard multidrug therapy (MDT) fails to achieve the desired response. By incorporating these alternative drugs, the study aims to improve treatment outcomes for nonresponsive cases of leprosy (Neto et al., 2020). Researchers and patients are increasingly exploring the use of plant-based drugs to address these challenges and achieve the complete eradication of the disease.

## 6 Alternative treatments for leprosy using plant-derived remedies

Leprosy is a treatable disease, and it has been successfully eliminated from many countries where plant-based medications have played a significant role. This achievement is attributed to the abundant availability of traditional knowledge, the use of medicinal plants, and the implementation of effective and safe drug delivery systems, which are detailed below.

### 6.1 Ethnomedicinal-based remedies for leprosy

On Earth, there are approximately 250,000 higher plant species, with more than 80,000 of them having medicinal uses for various purposes (Gupta et al., 2014). The diverse medicinal properties of these plants have contributed significantly to developing an effective healthcare system (Yesmin et al., 2008). India, often called the world’s herbarium, has been a rich source of natural remedies (Ganjewala and Gupta, 2013). In many parts of the country, especially in tribal and rural areas, herbal medicine is a vital component of healthcare (Bhandirge et al., 2016).

**FIGURE 7:**
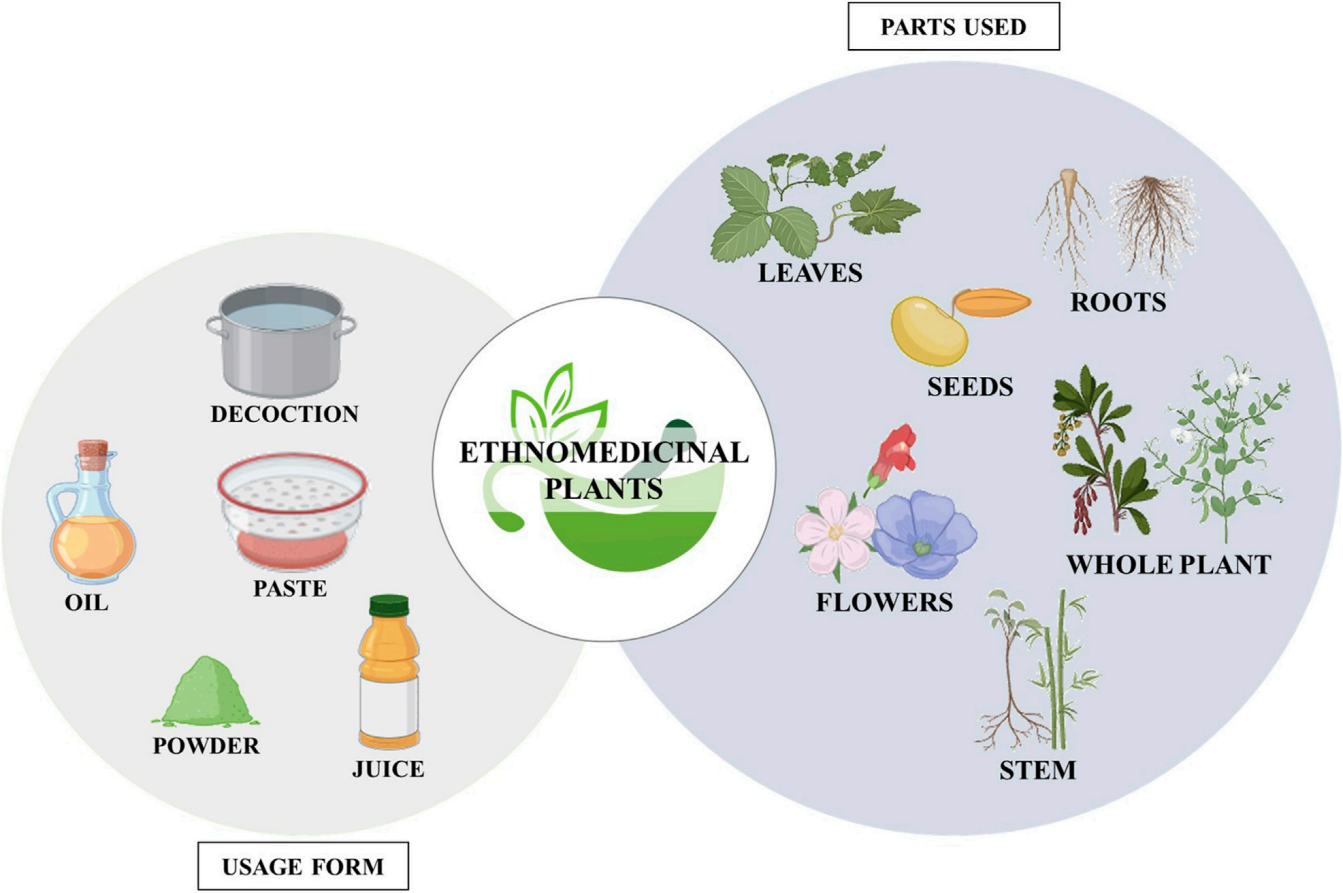
Diagram illustrating the plant parts utilized and the forms in which ethnomedicinal plants are used. (Biorender).

According to the WHO, approximately 80% of the population in developing countries rely on traditional medicine (Bhandirge et al., 2016). Plants have been crucial in sustaining and enhancing human life for thousands of years, providing valuable ingredients for beverages, cosmetics, medicines, and dyes (Azmi et al., 2011). At the community level, farmers, herbalists, healers, spiritualists, and hunters have been using traditional medicine as a primary healthcare system for centuries (Figure 7) (Mustapha et al., 2013). Table 1 lists the medicinal plants used by tribal people to treat leprosy.

**TABLE 1:** Ethnomedicinal plants employed by indigenous communities in the management of leprosy.

Botanical name	Common name	Family	Plant part used	Usage form	Location	References
*Abutilon indicum* G. Don	Pedipedika	Malvaceae	Leaves	-	Tamilnadu (Villupuram)	Mohanty et al. (2015)
*Achyranthes aspera* L	Naaiurvi	Amaranthaceae	Dried roots, Leaves	Powder	Andhra Pradesh (East Godavari), Assam (Morigaon)	Deka and Deka (2007)
*Ageratum conyzoides* L	Janglipudina	Asteraceae	Whole plant	Juice	Madhya Pradesh (Rewa), Haryana (Yamuna nagar)	Sahu et al. (2014)
*Amaranthus spinosus* L	Rangasuturia	Amaranthaceae	Roots, leaves	Paste	Assam (Morigaon)	Deka and Deka (2007)
*Anacardium occidentale* Linn	Jidimamidi	Anacardiaceae	Nuts, bark	Oil, bark powder mixed with honey	Andhra Pradesh (East Godavari), Tamilnadu (Kanyakumari)	Jeeva et al. (2007), Ramashankar and Sharma (2009)
*Andrographis paniculata* (Burm.f.) Wall. Ex. Nees	Kaalmegh	Acanthaceae	Whole plant, leaves	Paste	Odisha (Bhadrak), Bihar (Buxar)	Singh et al. (2013b), Panda et al. (2016)
*Angiopteris evecta* (Forst.) Hoff	-	Angiopteridaceae	Spores	-	Northeastern India	Benniamin (2011)
*Aquilaria malccensis* Lamk	Agaru	Linaceae	Whole plant	Oil	Assam (Morigaon)	Deka and Deka (2007)
*Argemone mexicana* L	Sialkata	Papaveraceae	Stem	Latex	Assam (Morigaon)	Deka and Deka (2007)
*Azadirachta indica* A Juss	Neem	Meliaceae	Seeds	Oil	Bihar (Buxar)	Sahu et al. (2014)
*Bauhinia variegata* L	Kachnar	Caesalpiniaceae	Root bark	Decoction	Uttarakhand (Udham Singh Nagar), Shimla Hills	Singh et al. (2014)
*Bidens pilosa* L	Ara–kajhar, Samsa	Asteraceae	Leaves	Paste	Bihar (Buxar)	Singh et al. (2013b)
*Borassus flabellifer* Linn	Tadi	Aracaceae	Fresh sap	Applied directly	Chhattisgarh (Dantewada)	Sahu et al. (2014)
*Butea monosperma* (Lam) Taub	Palas	Caesalpiniaceae	Flowers	Juice	Uttarakhand (Udham Singh Nagar), Odisha (Rayagada)	Kumar et al. (2012)
*Calatropis procera* W.T. Aiton	Aak, Akwan	Asclepiadaceae	Fruit, whole plant	Juice	Chhattisgarh (Raigarh)	Sahu et al. (2014)
*Calotropis gigantea* (L.) R. Br	Kempuyekke	Asclepiadaceae	Roots, bark, leaves and latex	Applied externally	Karnataka (Bidar), Madhya Pradesh (Balaghat)	Prashantkumar and Vidyasagar (2008), Jain et al. (2011), Sinha et al. (2013)
*Cassia fistula* Linn	Amaltas	Caesalpiniaceae	Roots, seeds, leaves	-	Chhattisgarh (Bastar)	Sahu et al. (2014)
*Celastrus paniculatus* Wild	Kunkunilata	Celastraceae	Roots, whole plant	Juice, decoction	Assam (Morigaon), Uttarakhand (Udham Singh Nagar)	Deka and Deka (2007)
*Centella asiatica* (L.)	Brahmi	Apiaceae	Whole plant	Decoction	Andhra Pradesh (East Godavari), Assam (Morigaon)	Deka and Deka (2007)
*Citrullus colocynthis* (L)	Verripuccha	Cucurbitaceae	Whole plant	-	Andhra Pradesh (East Godavari)	Divya et al. (2015)
*Commelina benghalensis* Linn	Konasimalu	Commelinaceae	Leaves, roots	Paste, powder	Assam (Morigaon), Haryana (Karnal)	Ravinder and Vashistha (2014)
*Corallocarpus epigaeus* Hk.f	Kurudankizhangu	Cucurbitaceae	Tubers	Boiled in coconut oil	Tamilnadu (Kanyakumari)	Jeeva et al. (2007), Kingston et al. (2009)
*Datura metel* L	Dhatura	Solanaceae	Roots	Paste mixed with neem oil	Chhattisgarh	Tiwari (2015)
*Dryopteris cochleata* (Ham ex D. Don) C. Chr	-	Dryopteridaceae	Rhizome	Powder	Northeastern India	Benniamin (2011)
*Eclipta alba* Hassk	Kaharaj	Asteraceae	Whole plant	Powder	Assam (Morigaon)	Deka and Deka (2007)
*Evolvulus alsinoides* Linn	Shankhyapuspi	Convolvulaceae	Whole plant	Extract	West Bengal (Bankura)	Sinhababu and Banerjee (2013)
*Holarrhena antidysenterica* (L.) Wall	Minamka chi	Apocynaceae	Whole plant	-	Meghalaya (Garo)	Deka and Deka (2007)
*Holarrhena pubescens* Wall. ex G. Don	Kurchi	Apocynaceae	Seeds	Decoction	Uttarakhand (Udham Singh Nagar)	Sharma et al. (2014)
*Indigofera aspalathoides* Vahl	Sivanarvembu	Papilionaceae	Bark	Powder	Tamilnadu (Kanyakumari)	Jeeva et al. (2007), Kingston et al. (2009)
*Ipomoea eriocarpa* R. Br	Nakhari	Convolvulaceae	Whole plant	Oil	Madhya Pradesh (Rewa)	Yadav et al. (2012)
*Jasminum grandiflorum* L	Jasmine	Oleaceae	Roots, leaves, flowers	-	West Bengal (Paschim Medinipur)	Hota and Chatterjee (2016)
*Jatropha goss ypifolia* L	-	Euphorbiaceae	Roots, leaves, seeds	-	Bihar (Buxar)	Kumari et al. (2016)
*Momordica charantia* L	Kerala	Cucurbitaceae	Fruits	Paste	Bihar (Buxar)	Deka and Deka (2007)
*Mussaenda frondosa* Linn	Mussanda	Rubiaceae	Roots	Paste	West Bengal (Bankura)	Sinhababu and Banerjee (2013)
*Ocimum sanctum* L	Tulasi	Lamiaceae	Leaves	Paste	Odisha (Bhadrak)	Deka and Deka (2007)
*Pongamia glabra* Vant	Koras	Papilionaceae	Seed	Powder, oil	Assam (Morigaon)	Deka and Deka (2007)
*Saussurea costus* (Falc.) Lipschitz	Kuth	Asteraceae	Roots	Paste	Himachal Pradesh (Lahaul-Spiti)	Lal and Singh (2008)
*Semecarpus anacardium* L.f	Bhilwa	Anacardiaceae	Gum	Applied directly	Uttarakhand (Udham Singh Nagar)	Sharma et al. (2014)
*Terminalia arjuna* (Roxb.ex DC.) Wight and Arn	Kawa	Combretaceae	Bark	Paste	Uttarakhand (Udham Singh Nagar)	Sharma et al. (2014)
*Terminalia bellarica* Roxb	Baheda	Combretaceae	Fruit	Dried powder	Maharashtra (Thane)	Sonawane et al. (2012)
*Terminalia catappa* Linn	Pateebadam	Combretaceae	Leaves	Juice	Andhra Pradesh (East Godavari)	Divya et al. (2015)
*Trichosanthes lobata* Roxb	Peppudal	Cucurbitaceae	Whole plant	Paste	Tamilnadu (Kanyakumari)	Yadav et al. (2012)

(- Not reported).

### 6.2 Pharmacological strategies utilizing plant-based compounds

Ethnobotanical and natural medicinal substances have the potential to significantly reduce the appearance of leprosy, and multiple studies have shown results comparable to approved treatments (Ruszymah et al., 2012; Sivaraj et al., 2017). Commercially available synthetic medications often come with various side effects that can worsen the condition of affected individuals, which has led to the growing popularity of plant-derived agents (Bhardwaj et al., 2016). Some herbal medicines also contain additional chemotherapeutic components for which there is no synthetic equivalent, further increasing the use of natural medicines. Phytomedicines have gained momentum due to public demand for herbal remedies and significant advancements in their potential therapeutic efficacy (Negi et al., 2012; Dubey and Kashyap, 2014). According to Singh et al., 2014, plant-based drugs are often used as a source of leprosy treatment because they are known to be beneficial in wound healing. Table 2 provides a list of plants with anti-leprotic properties along with pharmacological evidence.

**TABLE 2:** Pharmacological evidence supporting the antileprotic potential of plant extracts.

Botanical name	Plant part	Extract	Mode of administration	Dosage	Model organism	Novel outcome	References
*Abutilon indicum*	Roots	Ethanol	Incision, excision, and dead space wound model	400 mg/kg of the body weight	Albino rats	Increased wound contraction rate, skin breaking strength, and granuloma strength	Sohel (2015)
*Azadirachta indica*	Leaves	-	Excision and incision wound model	-	Sprague Dawley rats	Wound healing activity is facilitated by an enhanced inflammatory response and neovascularization	Alzohairy (2016)
*Azadirachta indica*	Seeds	Oil	Excision and incision wound model	-	Sprague Dawley rats	Significant dose dependent increase in wound healing	Alzohairy (2016)
*Bauhinia variegata*	Roots	Ethanolic and aqueous	Excision and incision wound models	200 and 400 mg/kg body weight	Albino Wistar rats	Significant dose dependent wound healing efficacy	Al-Snafi (2013)
*Calotropis procera* (Aiton) Dryand	Latex	-	Excision and incision wound and dexamethasone-suppressed wound healing rodent models	-	Guinea pigs	Single dose was significantly effective	Sharma and Kumar (2012)
*Centella asiatica*	-	Alcoholic, aqueous	Oral	-	Rats	Improvements in the symptoms of leprosy was observed	Brinkhaus et al. (2000)
*Chaulmoogra odorata* Roxb	Seeds	Chaulmoogra oil	Intraperitoneal route	Thrice a week	Wistar rats	Rapid wound healing was observed	Dey and De (2012)
*Hemidesmus indicus* (L.) R. Bx. ex Schult	Roots	Alcoholic	Applied as ointment	-	Mice	Significant change in the rate of wound contraction and epithelization duration	Chatterjee et al. (2014)
*Lanata camara* L	Leaves	Aqueous	Excision wound model	-	Male Sprague dawley rats	Greater wound contraction along with faster wound epithelization time	Nayak et al. (2009)
*Lanata camara*	Leaves	Ethanolic (5%, 10%)	Applied on neck lesions	-	Rats	Significantly higher wound healing	Saxena et al. (2012)

(- Not reported).

**FIGURE 8:**
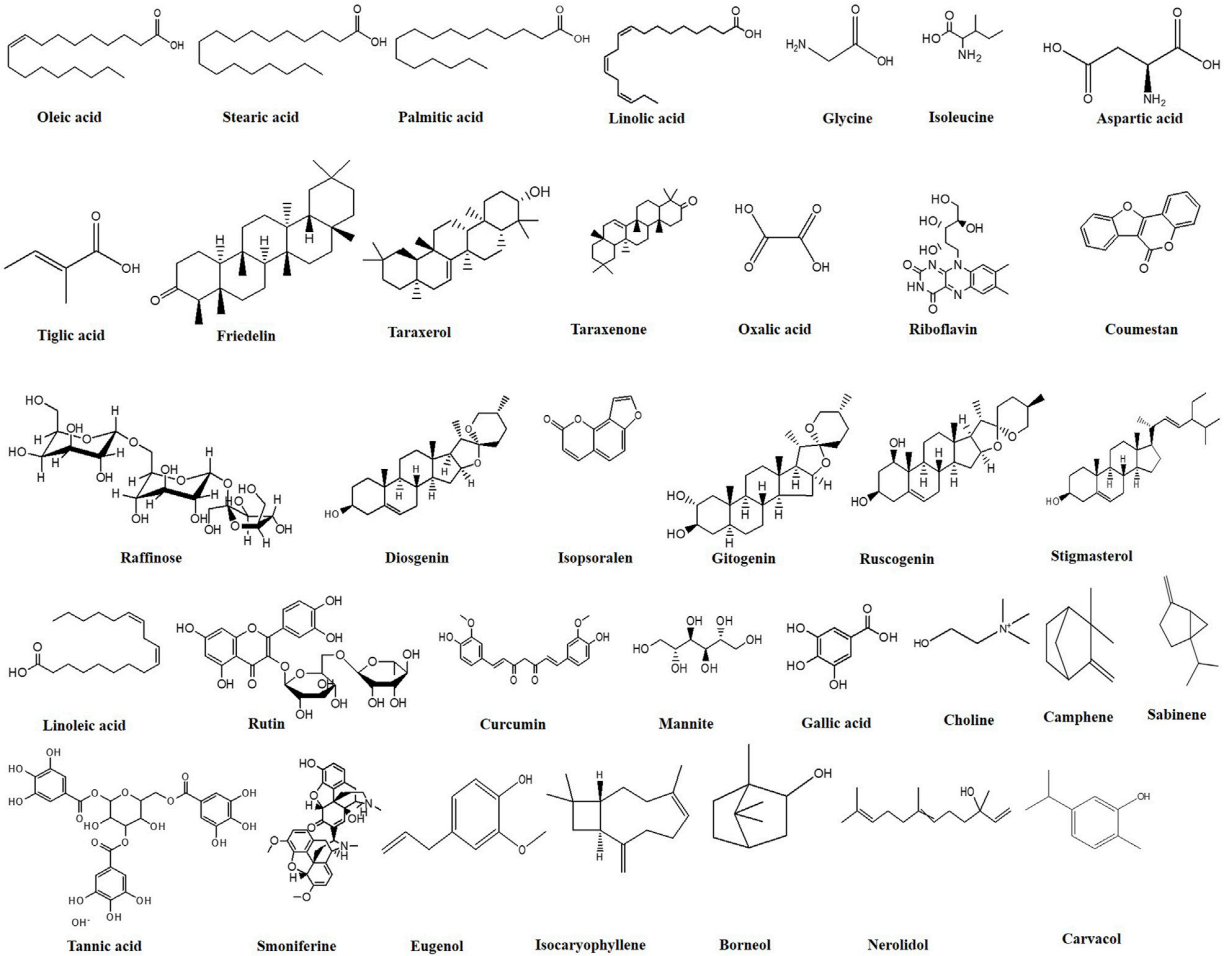
Chemical structures of secondary metabolites with anti-leprotic potential. (ChemDraw).

The chemical constituents with anti-leprotic activity isolated from *Centella asiatica* (*C. asiatica*) include saponin-containing triterpene acids and their sugar esters, particularly asiatic acid, madecassic acid and asiaticosides, such as asiaticoside, asiaticoside A, and asiaticoside B (Brinkhaus et al., 2000; Süntar et al., 2012). Chaulmoogra oil, one of the most widely used plant-derived anti-leprotic substances, is derived from the seeds of *Hydnocarpus wightiana* and *Chaulmoogra odorata*. According to Barbosa-Filho et al., 2007, the oil extracted from the seeds contains 90% esters of chaulmoogric acid and hydnocarpic acid, which are primarily responsible for anti-leprotic activity. *Abutilon indicum* is another phytomedicinal plant with the potential to treat various human ailments, primarily as an anti-inflammatory and anti-bacterial agent. Phytochemical analysis has revealed that the plant roots contain different nondrying oil-bearing fatty acids such as linoleic, oleic, stearic, palmitic, lauric, myristic, and capric acid. In contrast, the plant leaves contain amino acids, glucose, fructose, and galactose. According to Khadabadi and Bhajipale, stosterol and amyrin were also detected in the plant roots (Khadabadi and Bhajipale, 2010). Major bioactive metabolites with anti-leprotic potential are listed in Table 3, and their chemical structures are provided in Figure 8.

**TABLE 3:** Some secondary metabolites with anti-leprotic potential.

Botanical name	Common name	Secondary metabolites	References
*Amaranthus spinosus*	Spiny amaranth	β-sitosterol, stigmasterol, linoleic acid, rutin, catechuic tannins, carotenoids	Kumar et al. (2014)
*Bambusa arundinacea* Willd	Bamboo	Choline, betaine, cyanogenetic glycosides, albuminoids, oxalic acid, benzoic acid, arginine, cysteine, histidine, niacin, riboflavin, thiamine, glutelin, lysine, methionine	Sahu et al. (2014)
*Calotropis gigantea*	Giant milk weed	Stigmasterol, β-sitosterol, calotopin, uscharin, calotoxin, calactin, uscharidin, gigantin	Palejkar et al. (2012), Sinha et al. (2013)
*Clitoria ternatea* L	Butter fly pea, blue pea	Taraxerol, taraxerone, alkaloids, flavonoids, saponins, tannins	Sahu et al. (2014), Lijon et al. (2017)
*Curcuma longa* L	Turmeric	Curcumin, D-α-phellandrene, valeric acid, steroids, fatty acids	Mukesh et al. (2011)
*Eclipta alba*	False daisy, bhringraj	Coumestan, terpenoids, glycosides, sterols, alkaloids, sesquiterpenelactones, fatty alcohols, terthienylaldehyde, volatile oils, saponins, polyacetylinic compounds, flavonoids	Jahan et al. (2014)
*Ficus bengalensis* L	Banyan	Taraxasteroltiglate, methyl ether of leucoanthocyanidin, β-sitosterol, α-D-glucoside	Satyaveni et al. (2014)
*Ficus racemose* L	Country fig tree	Glauanol, hentriacontane, β-sitosterol, glauanol acetate, glucose, tiglic acid, esters of taraxasterol, lupeol acetate, friedelin, phytosterol	Sharma et al. (2012), Sharma and Kumar (2012), Deep et al. (2013)
*Garcinia morella* (Gaertn.) Desr	Gamboge	Morellin, neo-morellin, guttiferin	Sultana and Mukherjee (2015)
*Humulus lupulus* L	Common hop	Myrcene, humulene, beta-pinene, humuladienone, humulone, lupulone, beta-cubebene	Sultana and Mukherjee (2015)
*Lawsonia inermis* L	Henna	2-Hydroxynapthoquinine, mannite, tannic acid, mucilage, gallic acid	Mukesh et al. (2011), Sultana and Khosru (2011)
*Nerium indicum* Mill	Kaner	Neriodorin, neriodorein, karabin	Nagargoje and Phad (2003), Mukesh et al. (2011)
*Ocimum sanctum*	Tulsi	Camphene, citronellal, sabinene, limonene, ledol, eugenol, terpinolen, β-elemene, isocaryophyllene, iso-eugenol, α-amorphene, α-guaiene, α-humulene, α-terpineol, borneol, nerolidol, carvacrol, oleic acid, stearic acid, hexuronic acid, palmitic acid, linoleic acid, linolenic acid	Sahu et al. (2014), Bano et al. (2017)
*Psoralea corylifolia* L	Babchi	Essential oil, raffinose, psoralen, isopsoralen, caryophyllene, caryophyllin	Sultana and Mukherjee (2015)
*Senna occidentalis* L	Negro coffee, Kasundi	Oxymethylanthraquinone, glycine, histidine, isoleucine, leucine, aspartic acid, sitosterol, xanthone	Reeta and Ravindra (2013), Sahu et al. (2014)
*Tribulus terrestris* L	Gokharu, puncture vine	Diosgenin, gitogenin, chlorogenin, ruscogenin	Sultana and Mukherjee (2015)
*Withania somnifera* (L.) Dunal	Indian ginseng	Withanine, somniferine, somnine, somniferinine, withananine, pseudo-withanine, tropane, pseudo-tropine, choline, anaferine, anahydrine, isopelletierine, steroidal lactone	Rungsung et al. (2013), Bara et al. (2016)

*Bauhinia variegata*, a native medicinal plant, is highly valued for treating leprosy, particularly in India (Sharma and Kumar, 2012). This plant contains various bioactive metabolites, including flavonoids, steroids, terpenoids, tannins, lactones, glycolipids, glycosyl steroids, and quinines. Several flavonoids have been identified, such as rutin, apigenin, and apigenin 7-O-glucoside (Pachouri and Yadav, 2015). The stem bark of Bauhinia variegata also contains substances like kaempferol-3-O-D-glucoside, lupeol, β-sitosterol, 5,7-dihydroxy- and 5,7-dimethoxy flavanone-4-O-L-rhamnopyrosyl-D-glycopyranosides, and more (Al-Snafi, 2013).

#### 6.2.1 Mechanism of action

**FIGURE 9:**
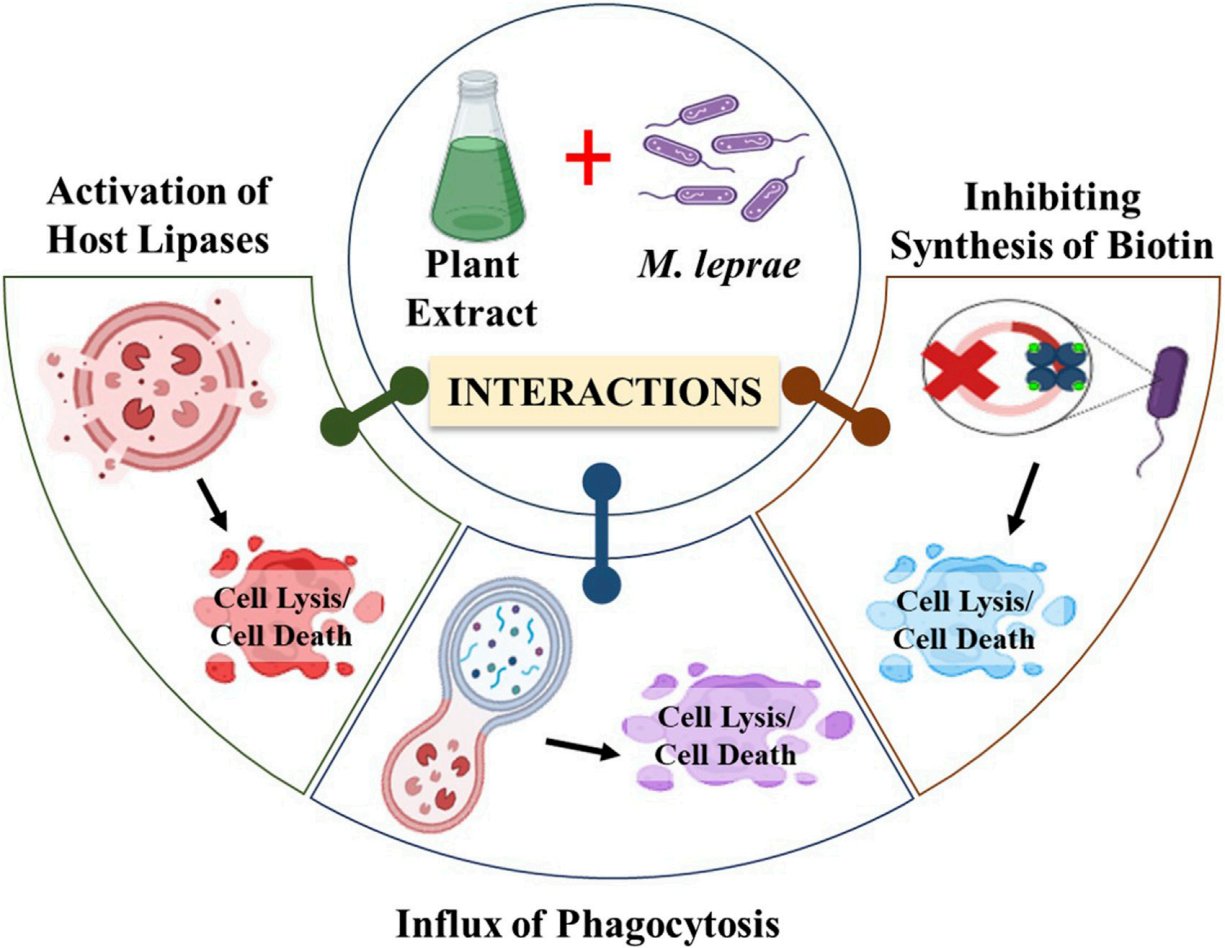
Diagrammatic representation of the mechanism of action of plant extracts against *M. leprae.* (Biorender).

Leprosy treatment often involves using various plant extracts and hydnocarpus oil, which has demonstrated positive outcomes. The potential anti-leprotic effects of these plant extracts and chaulmoogra oil may be attributed to multiple mechanisms. Firstly, they could trigger the activation of host lipases, enzymes responsible for breaking down foreign lipids, including the plant extract or chaulmoogra oil and the cell wall of *M. leprae* (Sahoo et al., 2014). Once the cell wall is compromised, the bacterium is believed to become susceptible to regular immune defense mechanisms, leading to its elimination (LeClere, 1996). Another suggested mechanism involves chemotaxis, where chaulmoogra oil may promote the recruitment of phagocytes to the vicinity of the bacterium, aiding in its clearance (Figure 9) (Norton, 1994). These mechanisms collectively contribute to the anti-leprotic effects of plant extracts and chaulmoogra oil.

Moreover, it has been proposed that cyclopentenyl fatty acids might exert their effects by inhibiting microbial biotin synthesis or interfering with biotin’s co-enzyme activity. Supporting this notion is the observation that a double bond in the cyclopentene ring appears essential for their antibacterial activity, as saturation of these double bonds diminishes their antibacterial properties (Jacobsen and Levy, 1973). Oommen et al. suggested that this double bond in cyclopentenyl fatty acids contributes to chaulmoogra oil’s anti-inflammatory and antioxidant characteristics. According to their hypothesis, the highly lipophilic long-chain carboxylic acids could displace the mycolic acid layer on mycobacteria, resulting in cell death (Oommen et al., 1999).

However, the stringent regulatory demands associated with using plant-derived substances as primary components in any formulation impose certain limitations on using these natural remedies. Therefore, it is imperative to emphasize the importance of assessing these natural agents’ risk-benefit ratio, safety, and toxicity profiles to enhance their availability to the wider population. Extensive research efforts should be dedicated to exploring potential metabolites as part of a concerted strategy to reduce the global impact of leprosy.

## 7 Nanotechnology’s emergence as a promising theranostic approach for leprosy

Despite the effectiveness of MDT in treating leprosy, there has been a persistent issue of low patient compliance with the medication regimen due to the unfavorable side effects associated with drugs like Dapsone, Rifampicin, and Clofazimine (Chakraborty and Biswas, 2020). These drugs fall into Class II category of the Biopharmaceutics Classification System, primarily due to their low solubility in water and challenges in oral drug absorption (Schneider-Rauber et al., 2020). In particular, Clofazimine is known to crystallize in tissues, including the intestinal lumen, due to the high dosage administered (Joshi et al., 2021). This has led to antibiotic resistance, particularly in countries like Brazil, India, and Southeast Asian nations, which contribute significantly to the global burden of leprosy. Addressing this challenge requires the development of innovative approaches to leprosy treatment (De Jong and Borm, 2008; Chenthamara et al., 2019). Several research teams are working on various nano-systems to enhance targeted drug delivery. These nano-systems can improve drug bioavailability, facilitate sustained drug release at the intended site, and reduce the required dosage. (Han et al., 2019; Kolahalam et al., 2019). Importantly, they also aim to mitigate the adverse effects associated with these medications, which can markedly improve patient compliance (Li et al., 2019; Mabrouk et al., 2021).

Chaves et al. developed an innovative approach to effectively deliver anti-leprotic medication for the treatment of leprosy using nano formulations. They created nanoparticles loaded with Dapsone (Dap), a key drug for leprosy treatment. These nanoparticles were designed to be pH-sensitive and were made using a copolymer called Eudragit L100 (EL100). To optimize the size of these nanoparticles, the researchers employed the Plackett Burman and Box-Behnken designs. The resulting nanoparticles had a size of approximately 198 nm. Importantly, these nanoparticles exhibited a unique drug release pattern. Most of the drug was released in a simulated stomach environment whereby the pH reached 6.8, although some remained encapsulated. This design allowed for prolonged drug release with enhanced permeability compared to the free drug. Furthermore, the Dap-loaded nanoparticles were non-cytotoxic to various cell lines, even at 400 g/mL concentrations. This suggests the great potential of these nanoparticles to serve as a safe and effective drug delivery system for leprosy treatment (Chaves et al., 2017).

The same research team also developed a nano-delivery system for Clofazimine (Clz) using a poly (D,L-lactide-co-glycolide; PLGA copolymer) based on a Plackett Burman Design. This system resulted in nanoparticles with a similar size to free Clofazimine (approximately 211 nm). The properties of the PLGA copolymer likely contributed to the advantages observed in this system. The degradation of the PLGA matrix enabled prolonged drug release, enhancing the therapeutic potential (Chaves et al., 2017). Combining these two nano-systems, Clz-PLGA and Dap-EL100, demonstrated effective intestinal permeability. Such a combination could improve patient compliance by reducing the toxicity of free drugs and minimizing intestinal damage (Chaves et al., 2018b). Chen et al. (2018) developed mesoporous silica nanoparticles with high Clofazimine loads using acetophenone as a solvent. These nanoparticles achieved high levels of Clofazimine delivery, enabling bacteria killing without harming macrophages (Chaves et al., 2018b). Several anti-leprotic medications have been successfully delivered using solid-lipid nanoparticles (SLNs) (Table 4). Reis and coworkers utilized the Box Behnken design to optimize mannosylated SLNs with a particle size of approximately 333.2 nm (Kanwar et al., 2018). The release rate of Dapsone from mannosylated SLNs was found to depend on pH, with faster release at acidic pH levels than at neutral pH. Additionally, these nanoparticles targeted intestinal microfold cells (M-cells), which mannosylate to facilitate antigen absorption from the intestinal lumen by the immune system. These nanoparticles remained stable for 8 weeks (Vieira et al., 2016). Kanwar et al., 2018 developed SLNs loaded with Dapsone and Rifampicin using another class of SLNs called lactonic sophorolipids, stabilized with various non-ionic polymeric surfactants (Pluronic F68 and F127). They predicted the drug release profile kinetically. The drug transport mechanisms for Dapsone and Rifampicin were determined to be Fickian-driven and Non-Fickian, respectively. Despite the rapid drug release from the SLNs, the drug concentrations remained within therapeutic limits, indicating controlled drug release (de Almeida Borges et al., 2013).

**TABLE 4:** Reports on nanoformulations for targeted delivery of anti-leprotic drugs and leprosy treatment.

Nanoparticles	Size (nm)	Drug	Dosage	Mode of action	Novel outcomes	References
Eudragit L100 nanoparticles	198 ± 6	Dapsone	400 μg/mL	Sustained pH sensitive drug release at pH above 6	Nanoparticles were able to protect the cells from adverse effect of drugs	Chaves et al. (2017)
Lactonic sophorolipids polymeric surfactant	136	Rifampicin	-	Non-Fickian process	Increased drug solubility and permeability, resulting in increased medication bioavailability	Kanwar et al. (2018)
Lactonic sophorolipids polymeric surfactant	144	Dapsone	-	Fickian driven process	Improved bioavailability, reduced toxicity, controlled drug release	de Melo Barbosa et al. (2021)
Mannosylated Solid Lipid	333.2 ± 2.3	Dapsone	-	pH sensitive drug release (more efficient at lower pH)	Faster drug release	Vieira et al. (2016)
Mesoporous silica nanoparticles	-	Clofazimine	0.5, 1 and 2.0 μg/mL	Acetophenone assisted clofazimine drug delivery	Significant enhancement in drug release efficiency	Chen et al. (2018)
Nanoemulsion	-	Dapsone	-	Oil in the water system leading to drug release	Reproducible bioavailability and a constant plasma con-centration profile	Monteiro et al. (2012)
Nanogold and silver nanoparticles	-	MDT	-	Reduces the wound width by 80% in patients under the MDT regimen	Significantly higher efficacy	Taufikurohmah et al. (2019)
PLGA	211 ± 3	Clofazimine	-	Sustained drug release after degradation of PLGA matrix	Controlled drug release	Chaves et al. (2018a)
Topical nanoemulsion	-	Dapsone	-	Higher epidermal penetration, higher drug release rate	Permeation of drug through barrier	de Almeida Borges et al. (2013)

(- Not reported).

Nanoemulsions from micellar solutions serve as effective drug delivery systems that protect pharmaceutical medications from hydrolysis and degradation, thereby enhancing drug absorption (Monteiro et al., 2012; Islan et al., 2017). Dapsone-containing topical (de Almeida Borges et al., 2013) and oral nanoemulsions were developed (Monteiro et al., 2012). In the case of the Dapsone oral nanoemulsion, it employed a water-in-oil (w/o) system that was converted *in vivo* into an oil-in-water (o/w) system by incorporating propylene glycol as a co-solvent. This inversion process increased drug permeability and dissolution compared to Dapsone in powder form (Monteiro et al., 2012). Topical administration of the drugs helped minimize adverse effects associated with higher doses (de Almeida Borges et al., 2013). To create a topical Dapsone nanoemulsion, isopropyl myristate and N-methylpyrrolidone were used. The latter exhibited greater Dapsone solubilization and *in vitro* release rate, while the former demonstrated better *in vitro* epidermal penetration. Both formulations remained stable for 3 months (de Almeida Borges et al., 2013). Taufikurohmah et al., 2019 utilized a combination of gold and silver nanoparticles to treat epidermal lesions related to leprosy topically. They observed that this combination effectively reduced wound width by 80% in patients undergoing MDT regimen, whereas the control group’s wound width had only decreased by 50%. However, the exact mechanism by which these nanoparticles inhibit the growth and survival of *M. leprae* remains largely unclear. The pathogen’s non-culturable nature in artificial medium has prompted suggestions that nanoparticles may target certain overexpressed antigenic proteins within the pathogen during infection (Chakraborty and Biswas, 2020). This research indicates that nano-formulations, which can specifically target significant mycobacterial proteins like HSP18, may hold a great promise for effective treatment of leprosy in the future.

### 7.1 Limitations of nanoparticles as potential drug delivery systems

Nanoparticles offer numerous advantages over conventional medicinal agents. However, their potential for clinical success is significantly hampered by nanotoxicity, particularly in the context of drug delivery processes. Some reports suggest cationic nanoparticles pose a greater risk than anionic nanoparticles (Chenthamara et al., 2019). Several inherent properties of nanoparticles, such as higher surface reactivity and a greater surface-to-volume ratio, contribute to their potential toxicity (Bahadar et al., 2016). The formation of a “protein corona,” which occurs when nanoparticles interact with various biomolecules, especially proteins, can alter the particles’ pharmacological behavior and potentially lead to toxicity (Lee et al., 2015). Reactive oxygen species (ROS) generated due to interactions between nanoparticles and the protein corona can cause cellular damage. Metal-based nanoparticles can also produce ROS through surface modulation as well as Fenton, Fenton-like, and Haber-Weiss reactions (Kessler et al., 2022). Numerous efforts have been made to evaluate the potential hazards of nanoparticles derived from various blood cell components (Magdolenova et al., 2015; Farace et al., 2016). Nanoparticle toxicity is also influenced by the size of the nanoparticles. Battal et al., 2015 reported that the genotoxic, mutagenic, and cytotoxic effects of SiO_2_ nanoparticles are significantly size-dependent. Surface coating also affects these nanoparticle impacts. Magdolenova et al., 2015 demonstrated that coated iron oxide nanoparticles are more cytotoxic and genotoxic than uncoated iron oxide nanoparticles. The biocompatibility of nanoparticles must be carefully assessed because they can induce toxicity when interacting with blood cells (Chakraborty et al., 2023). Additionally, conducting extensive research on how nanoparticles degrade within the body is crucial to mitigate their hazardous effects.

## 8 Conclusion and future perspective

Leprosy remains a significant public health concern in many developing countries, underscoring the importance of early diagnosis for effective control. This review provides insights into the use of synthetic drugs, medicinal plants, and recent advancements in nano-pharmaceutics for leprosy treatment. The growing interest in traditional medicine worldwide and its integration with pharmacological approaches have accelerated global pharmaceutical development. Nanotechnology and the use of nanomaterials have ushered in revolutionary changes in various fields of medical research. The convergence of ethnopharmacology and nanotechnology offers a promising paradigm for advancing antileprotic research and treatment strategies. By harnessing the synergies between traditional knowledge and modern science, we can accelerate progress towards achieving the ultimate goal of eliminating leprosy as a public health concern.The review suggests that nanoparticles hold promise for targeted drug delivery in leprosy treatment, possibly due to their ability to enhance the permeability of mycolic acid cell walls. However, comprehensive research efforts are needed to develop biodegradable nanomaterials that can efficiently deliver various disease-modifying drugs, ultimately leading to the successful treatment of leprosy. It is worth noting that nanoparticles exhibit stronger antibacterial potential compared to plant extracts. Therefore, exploring the synthesis of nanoparticles from plant extracts with anti-leprotic properties could be a promising avenue. Additionally, assessing the biodegradability and cytotoxicity of nanoparticles is crucial due to their potentially harmful effects. These forward-looking approaches focus on developing safe, biocompatible, effective, target-specific, and reliable nanoparticles with improved pharmacokinetic behavior towards the treatment of leprosy. Future research should focus on exploring nanotechnological innovations for enhancing the delivery, efficacy, and stability of antileprotic agents. Tailoring nanoparticle characteristics such as size, surface charge, and targeting ligands can optimize drug pharmacokinetics and tissue distribution. Furthermore, the potential synergistic effects of combining traditional remedies with conventional antileprotic drugs or nanotechnology-based formulations. Combination therapies may enhance efficacy, reduce drug resistance, and minimize adverse effects, paving the way for more personalized treatment regimens.

## Abbreviation

A-MDT, Accompanied-multi drug therapy; BB, Borderline-borderline leprosy; BL, Borderline lepromatous leprosy; BT, Borderline tuberculoid leprosy; CD, Cytotoxic T-cells; CMI, Cell-mediated immune response; CR, Complement receptor; EL100, Eudragit L100; ENL, Erythema nodosum leprosum; FQ, Fluoroquinolone; GLP, Global leprosy program; IFN, Interferon; IL, Interleukin; LL, Lepromatous leprosy; M, Macrophages; *M. leprae*, *M. leprae*; MB, Multibacillary individuals; MDT, Multidrug therapy; NFAT, Nuclear factor of activated T-cells; PGL-I, Phenolic glycolipid I; PGLA, Poly (glycolide-co-lactide); PNL, Pure neural leprosy; PPD, Purified protein derivative; ROS, Reactive oxygen species; RR, Reversal responses; SC, Schwann cell; SLNs, Solid lipid nanoparticles; TGF-β, Transforming growth factor-beta; Th, T helper cells; TLR, Toll-like receptors; TNFα, Tumor necrosis factor-alpha; TT, Tuberculoid leprosy.
